# An investigation of the middle and late behavioural phenotypes of Mucopolysaccharidosis Type-III

**DOI:** 10.1186/1866-1955-6-46

**Published:** 2014-12-31

**Authors:** Elaine M Cross, Sheena Grant, Simon Jones, Brian W Bigger, James E Wraith, Louise V Mahon, Michelle Lomax, Dougal J Hare

**Affiliations:** Section for Clinical and Health Psychology, School of Psychological Sciences, University of Manchester, Zochonis Building, Brunswick Street, Manchester, M13 9PL UK; Department of Genetic Medicine, St Mary’s Hospital, Manchester, UK; Stem Cell & Neurotherapies, Faculty of Medical and Human Sciences, University of Manchester, Manchester, UK

**Keywords:** Sanfilippo syndrome, Mucopolysaccharidosis, MPS III, Behavioural phenotype

## Abstract

**Background:**

Mucopolysaccharidosis type-III (MPS III) is an autosomal recessive lysosomal storage disorder. It causes progressive physical and cognitive decline and has been linked to increased incidences of behavioural problems.

**Methods:**

Data on the behaviour and adaptive skills of 20 children with MPS III and 25 children with intellectual disability (ID) (17 included in analysis) were gathered via parental report questionnaire. The frequencies of different types of behaviour displayed by children with MPS III and children with ID were compared across two age categories.

**Results:**

The total frequency of challenging behaviours displayed by children aged 2–9 years with MPS III and ID was not significantly different. Behaviours associated with hyperactivity, orality, unusual body movements and inattention were seen significantly more frequently in 2–9 year olds with MPS III than in those with ID. Children aged 10–15 years with MPS III showed significantly fewer problem behaviours than a contrasting group with ID. The frequency of challenging behaviours displayed by children with MPS III and their adaptive skills was found to decrease with age.

**Conclusions:**

Behaviours relating to hyperactivity, orality, unusual body movements and inattention are part of the behavioural phenotype of the middle phase of MPS III. The late phase of MPS III is associated with low rates of problem behaviour and loss of adaptive skills. Therefore, families with a child with MPS III may benefit from a different type of clinical service when the child is aged 2–9 years, than when aged 10–15 years.

## Background

Mucopolysaccharidosis type-III (MPS III (Sanfilippo syndrome)) is a recessively inherited lysosomal storage disorder and is the most prevalent of the seven mucopolysaccharide (MPS) disorders, occurring 0.28–4.1 in 100,000 live births [1]. MPS disorders are caused by deficiency in enzymes responsible for the degradation of glycosaminoglycans (GAGs) and subsequent GAG accumulation in various organs causes a multi-system disease [2]. MPS III has four subtypes A to D associated with a specific enzyme deficiency. All four enzymes, heparan N-sulfatase, a-N-actylglucosaminidase, acetyl-CoA: a-glycosaminide and N-acetylglucosamine 6-sulfatase (A to D, respectively), are associated with the breakdown of heparan sulphate [3]. The most prevalent type in the UK is type A; type B is less common and types C and D rare [4].

MPS III causes severe neurological impairment and a gradual decline in functioning with a tri-phasic clinical course. The beginning phase (1–2 years) is characterised by developmental delay but normal stature and physical growth [5]. The middle phase (2–9 years) shows considerable variation and is characterised by behavioural problems and sleep disturbance. The late phase (10+ years) is associated with skill loss, reduced behaviour problems, loss of motor skills, increased spasticity, seizures and swallowing difficulties [5]. Other symptoms include recurrent diarrhoea; ear, nose and throat infections; and visual impairment [6]. Age of death varies within and between subtypes with a median of 15.2 years for type A [7] and 34 years for type C [8].

A recent survey of care professionals and families investigating non-carcinomatous life-limiting conditions identified MPS disorders as the primary priority for further research, given the complex symptom profile, difficulties in managing symptoms and distress experienced by families [9, 10]. Research into treatments is ongoing but inconclusive [11].

A recent systematic review of behaviour and development in MPS III [12] identified behaviour problems, including restlessness and hyperactivity, physical aggression, unusual affect (laughing/screaming/crying), ‘tantrums’ and orality [5, 7, 8, 13–20], as strongly associated with the middle phase, thence declining with age and loss of functions [8]. Sleep and circadian rhythm were found to be significantly different from matched controls in two studies [5, 21]. Linguistic and motor development was ‘relatively normal’ for the 1st year with first signs and symptoms differing between subtypes, ranging from 2 years 3 months to 5 years. Age at onset of cognitive delay and rate of decline increased across types A to D respectively [13].

Research to date has been limited by inadequate measurement, control groups, statistical analyses and methodologies (e.g. case-note review). To address this, the present study used validated and syndrome-specific measures and a genetically distinct, ability-matched, control group to address the following research questions:

1: Do the frequencies of challenging behaviour differ significantly between children with MPS III and children with ID?

2: Are any types of challenging or adaptive behaviour observed significantly more frequently in children with MPS III than in children with ID?

## Methods

### Recruitment

#### Children with MPS III

This study was conducted alongside other studies investigating sleep, circadian rhythm and family functioning [22, 23] with recruitment through the MPS Society UK and a genetics department in the North West of England. Questionnaires were sent to 25 families with a child with MPS III with 20 returned.

#### Children with intellectual disability (ID)

Families of children with intellectual disability (ID) were recruited through national and local MENCAP and 30 local parent support agencies across the UK. Sixty-six questionnaire packs were sent out with 24 returned.

### Sample

Children with MPS III were included in the study if they had a diagnosis of MPS III (any subtype) made via genetic/enzyme testing, were resident in the UK and their parents understood written English. People with MPS III were excluded if they had received gene or enzyme replacement therapy or a bone marrow transplant and if they were under 2 years of age. Children with ID were included if they had an intellectual disability, were aged 2–15 years, their parents understood written English and were resident in the UK and were excluded if they had an autistic spectrum condition but an IQ > 70 and if they were under 2 years of age.

### Design

Parents/carers (MPS III or ID) ‘opted in’ via telephone or email. Information and consent forms and questionnaires were sent via post. When possible, families were telephoned to collect missing data.

### Materials/measures

Demographic Questionnaire—used to collection information on age, diagnosis, treatments received, deafness, blindness, epilepsy, medications and GP details.

Learning Disability Casemix Scale (LDCS) [24]—measures degree (mild/moderate/severe) of ID (A) and frequency and severity of challenging behaviour (C), based upon the widely used Wessex Behaviour Schedule [25].

Vineland Adaptive Behavior Scale, Second Edition-Parent/Carer Rating Form (VABS-II) [26]—measures current adaptive and maladaptive behaviour across 11 subdomains within 4 domains of *communication*, *daily living skills*, *socialisation* and *motor skills*. Each subdomain contains lists of adaptive skills and respondents rate if the child/adult can do this; ‘Usually’ = 2, ‘Sometimes/Partially’ = 1 or ‘Never’ = 0. The measure gives an overall adaptive behaviour score (*Adaptive Behaviour Composite*) as well as age equivalent scores and standard scores for each domain. Internal consistency reliability is moderate to high for domain scores (*a* = 0.71–0.95) and high for *Adaptive Behaviour Composite* score (*a* = 0.86–0.98) across all ages [26].

Aberrant Behaviour Checklist (ABC) [27]—measures severity of a child’s behaviour in the last month, with each behaviour problem rated from 0 (not a problem at all) to 3 (the problem is severe in degree) across domains of *irritability/agitation*, *crying/lethargy*, *social withdrawal/stereotypic behaviour*, *hyperactivity/noncompliance* and *inappropriate speech*. Internal consistency is good across all domains (*a* = 0.86–0.95) [27–29].

*Eyberg Child Behaviour Inventory* (*ECBI*) [30, 31]—measures frequency and severity of current behavioural problems for children aged 2–17 years, with frequency of behaviours rated from 1 (*never*) to 7 (*always*) to give a behaviour ‘*intensity*’ score. Respondents state if each behaviour is a problem for them, and the number of problematic behaviours is summed to give a ‘*problem*’ score. The ECBI has high internal consistency for both *problem* (*a* = 0.94) and *intensity* (*a* = 0.95) domains [32]. It has been found to provide a homogenous measure of conduct problems when used via post [32].

Sanfilippo Behaviour Rating Scale (SBRS) [33]—comprises three sections: *communication*, *tantrums* and *behaviour*. The scale is composed of past and present communication skills (Section I); frequency, duration and emotions expressed during tantrums (Section II); and frequency, onset and cessation of relevant motor, perceptual, social and emotional skills and behaviour (Section III). The SBRS is under development for use in MPS III treatment trials.

### Statistical analysis

All data were anonymised, stored and analysed in accordance with the Data Protection Act (1998). Data were analysed using the Statistical Package for the Social Sciences (SPSS) versions 16.0 and 19.0. Children were divided into age groups associated with stages of the disorder: 2–9 years (middle phase) and 10–15 years (late phase), with poorly matched controls being excluded from the ID group.

Questionnaire scores were tested for normality using the Kolmogorov-Smirnov test and by examination of Q-Q and P-P probability plots (graphical representation and comparison of the data distribution). Although most scores were normally distributed, the sample size was small, and non-parametric statistics (Mann-Whitney U and Spearman rho) were used for all analyses with two-tailed significance values. As the SBRS is a relatively new measure, Cronbach’s *α* was calculated to test for reliability (internal consistency).

Total measure scores and domain scores were calculated according to the measure guidelines. The functioning of children with MPS III and ID was so low that the standardised scores and some age-equivalent scores on the VABS-II were not meaningful, and raw scores were therefore used for comparison as the groups were matched for age and ability. Raw scores were summed to give domain raw scores, and these were summed to give a measure raw score. All measure scores, domain scores and subdomain scores were compared between children with MPS III and children with ID. Bonferroni adjustments were not used as these would have given too conservative a cut-off for significance, increasing the chance of Type II errors [34]. Effect sizes (*r* = *Z*/√*n* [35]) were computed for all significant findings taking *p* < 0.05 used as cut-off for significance in all comparisons.

### Ethical approval

This study was approved by NHS North West Research Ethics Committee, University of Manchester School of Psychological Sciences Ethics Committee and Central Manchester Foundation Trust Research and Design department.

## Results

Data for 20 children with MPS III (*N* = 10 aged 2–9 years; *N* = 10 aged 10–15 years) and 25 children with ID (*N* = 15 aged 2–9 years; *N* = 10 aged 10–15 years) were collected. In the 2–9-year age group, all children with MPS III had severe ID, and therefore, only children receiving a score indicative of severe ID were included in the control group (*N* = 10 remained). In the 10–15-year age group, all the children with MPS III had severe or moderate ID and children with mild ID were excluded from the comparison group (*N* = 7) (Table 1).

**Table 1:** **Participant demographics**

		2–9-year age group	10–15-year age group	16+ year age group
MPS III	*N*	10	10	5
	Median age	4.5	12.5	28
	Youngest to oldest	2–9	10–15	16–32
	Median ID score	30 (*N* = 8)	37.5	39
	(range)	(26–36)	(20–40)	(31–41)
	Gender	7 male, 3 female	4 male, 6 female	2 male, 3 female
	Genetic subtypes	2xA, 7xB, C	7xA, 3xB	2xA, 2xB, C
ID	*N*	10	7	-
	Median age	4	12	
	Youngest to oldest	2–8	10–15	
	Median ID score	31.5	22	
	(range)	(26–38)	(17–32)	
	Diagnosis	2xASD, 3xDS, AS, CD	3xASD, AS, CD	
	Gender	7 male, 3 female	4 male, 3 female	

*ASD* autism spectrum disorder, *DS* Down syndrome, *AS* Angelman syndrome, *CD* chromosome deletion [unspecified].

The SBRS *current understanding*, *past understanding*, *orality*, *body movements*, *fearfulness*, *attention*, *self-control/compliance* and *mood*, *anger and aggression* domains had good internal reliability (*α* > 0.7), the remaining domains having poor internal reliability (*α* < 0.7).

**Figure 1:**
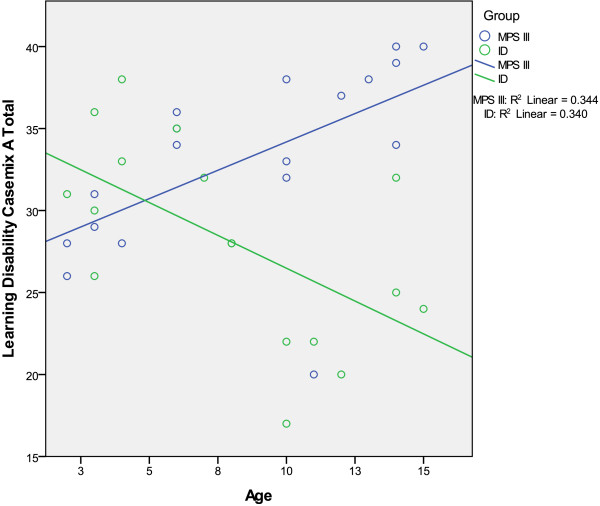
**Graph showing the relationship between age and disability score.**

As seen in Figure 1, there was an outlier in the MPS III group with a high level of skills aged 11 years. Subsequent analyses were conducted both with and without this outlier, but the latter are only reported if these differed from those conducted with the whole dataset. Skills increased with age for the ID group (green line) but decreased with age for the MPS III group (blue line), with LDCS A score being significantly correlated with age in the MPS III group (*r* = 0.728, *p* = 0.01).

Frequency of challenging behaviour (ECBI Intensity score) and level of disability (LDCS A score) were negatively correlated in both the MPS III (*r* = -0.676, *p* = 0.008) and ID (*r* = -0.573, *p* = 0.02) groups, but this relationship was non-significant in the MPS III when the outlying case was omitted, which was most likely due to the lack of variability in the MPS group.

**Figure 2:**
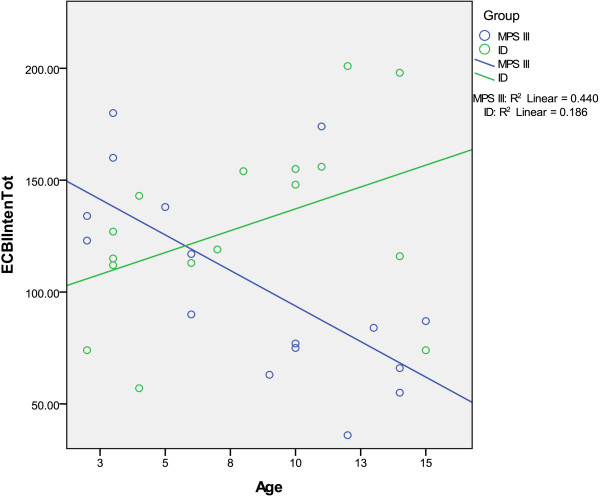
**Graph of the relationship between ECBI Intensity score and age.**

Figure 2 shows the relationship between the ECBI behaviour *intensity* score and age. For the MPS III group (blue line), the frequency of behavioural problems reduced with age, while for the ID group (green line) the frequency increases. Age and *intensity* score were significantly negatively correlated for children with MPS III (*r* = -0.639, *p* = 0.008), but this was non-significant when the outlier was removed.

### Middle phase (2–9-year-old group)

In terms of adaptive skills measured by the VABS-II, MPS III group scores were significantly higher than ID scores for the *gross motor skills* subdomain only, with a large effect size (*U* = 13, *z* = -2.493, *r* = -0.605, *p* = 0.013) (Tables 2 and 3).

**Table 2:** **VABS-II subdomain scores (2–9 year olds)**

Domain	Subdomain	MPS III raw score median ( ***N***, range)	MPS III age equivalent (years: months)	ID raw score median ( ***N***, range)	ID age equivalent (years: months)	***p***value
Communication	Receptive	11 (9, 5–16)	1:0	12.5 (10, 6–23)	1:1	0.512
	Expressive	17 (9, 9–42)	0:10	13 (10, 8–22)	0:8	0.164
	Written	0 (8, 0–3)	≤1:10	0 (9, 0–14)	≤1:10	0.301
Daily living skills	Personal	15 (9, 7–26)	1:5	12 (10, 4–34)	1:2	0.870
	Domestic	1 (9, 0–10)	0:10	0 (10, 0–8)	≤0:7	0.435
	Community	3 (9, 0–7)	0:11	3 (10, 0–8)	0:11	0.901
Socialisation	Interpersonal relationships	23 (8, 15–36)	0:9	22.5 (10, 14–43)	0:8	0.447
	Play and leisure	13.5 (8, 0–26)	1:2	6 (9, 3–22)	0:7	0.311
	Coping skills	5 (9, 0–18)	1:1	5.5 (0, 0–13)	1:1	0.967
Motor skills	Gross	59 (7, 46–62)	2:5	42 (10, 12–58)	1:4	0.013
	Fine	30 (7, 14–37)	2:8	23 (9, 9–28)	2:0	0.050

**Table 3:** **Behaviour-related domain scores (2–9 age group)**

Measure/domain	MPS III	ID	***p***value
	**Median (** ***N*** **, range)**	**Median (** ***N*** **, range)**	
ECBI			
Intensity score	128 (8, 63–180)	115 (9, 57–154)	0.336
Problem score	16 (7, 0–27)	11 (9, 1–20)	0.761
ABC			
Irritability	8 (7, 3–31)	11.5 (10, 1–22)	0.494
Lethargy	10 (7, 0–29)	9.5 (10, 1–29)	0.732
Stereotypy	4 (7, 0–12)	0.5 (10, 0–14)	0.577
Hyperactivity	27 (7, 10–38)	11 (10, 5–28)	0.031
Inappropriate speech	3 (7, 0–9)	0 (10, 0–5)	0.08
ABC total score	58 (7, 13–113)	34 (10, 10–91)	0.525
SBRS			
Current understanding	28 (9, 11–35)	24.5 (10, 5–40)	0.902
Past understanding	30 (7, 24–41)	12.5 (4, 0–35)	0.130
Current expression	8 (9, 1–12)	6.5 (10, 4–14)	0.967
Past expression	6 (6, 2–17)	3 (5, 0–6)	0.125
Orality	29 (9, 11–33)	12.5 (10, 0–24)	0.005
Body movements	22 (9, 5–27)	6.5 (10, 0–18)	0.013
Interactions with objects	14 (9, 2–20)	**7** (10, 0–12)	0.022
Activity and routines	22 (9, 11–36)	15.5 (10, 8–26)	0.078
Emotional function	5 (9, 0–16)	4 (10, 0–12)	0.536
Safety consciousness	14 (9, 8–18)	10.5 (10, 6–18)	0.267
Fearfulness	28 (9, 16–38)	28 (10, 9–40)	0.806
Social interaction	16 (9, 8–24)	20 (10, 12–26)	0.388
Eye contact	8 (9, 2–18)	5 (10, 0–10)	0.201
Emotional engagement	7 (9, 0–13)	11.5 (10, 1–16)	0.234
Comfort seeking	9 (9, 6–23)	5.5 (10, 0–24)	0.078
Attention	14 (9, 10–18)	8.5 (10, 5–18)	0.040
Self-control/compliance	11 (9, 0–18)	9.5 (10, 2–15)	0.461
Mood, anger and aggression	11 (9, 5–33)	10 (10, 4–24)	0.582
Self-gratification	0 (9, 0–7)	0.5 (10, 0–11)	0.649

In the MPS III group, the median ECB score in the MPS III group exceeded clinical cut-off (15); ABC *hyperactivity* scores were significantly higher with a large effect size (*U* = 13, *z* = -2.151, *r* = -0.522, *p =* 0.031), and SBRS domain scores were significantly higher for *orality* (*U* = 11, *z* = -2.78, *r* = -0.638, *p =* 0.005), *body movements* (*U* = 14.5, *z* = -2.493, *r* = -0.572, *p =* 0.013), *interactions with objects (U* = 14.5, *z* = -2.493, *r* = -0.572 *p =* 0.022) and *attention* (*U* = 20, *z* = -2.054, *r* = -0.471 *p =* 0.04) domains. Of the children with MPS III, 67% reported some sleep problems and 33% reported severely disrupted sleep.

### Late phase (10–15 years group)

Total VABS-II measure and domain scores were lower in the MPS III group with *daily living skills* being significantly so (*U* = 8.5, *z* = -2.261, *r* = - 0.584, *p* = 0.024) (Table 4). Significantly lower scores with large effect sizes were reported for w*ritten communication* (*U* = 11, *z* = -2.042, *r* = -0.527, *p* = 0.041), *personal skills* (*U* = 9.5, *z* = -2.143, *r* = -0.553, *p* = 0.032), *domestic skills* (*U* = 3, *z* = -3.05, *r* = -0.788, *p* = 0.002), *community skills* (*U* = 8.5, *z* = -2.288, *r* = -0.591, *p* = 0.022) and *coping skills* (*U* = 9.5, *z* = -2.16, *r* = -0.558, *p* = 0.031) subdomains. When the outlier in the MPS III group was removed, significantly lower scores were reported for both *gross motor skills* (*p* = 0.018) and *fine motor skills* (*p* = 0.030). All age-equivalent scores for children with MPS III fell below 18 months.

**Table 4:** **VABS Subdomain scores (10–15 year olds)**

Domain	Subdomain	Median MPS III score median ( ***N***, range)	MPS III age equivalent (years: months)	Median ID score median ( ***N***, range)	ID age equivalent (years: months)	***p***value
Communication	Receptive	10.5 (8, 3–33)	0:11	21 (7, 8–28)	1:9	0.223
	Expressive	16 (8, 2–90)	0:9	61 (7, 9–73)	2:10	0.165
	Written	0 (8, 0–21)	≤1:10	10 (7, 0–41)	4:6	0.041
Daily living skills	Personal	11 (8, 0–58)	1:1	40 (7, 14–52)	2:11	0.032
	Domestic	0 (8, 0–8)	≤0:7	13 (7, 1–22)	4:11	0.002
	Community	1 (8, 0–24)	0:3	22 (7, 3–23)	4:10	0.022
Socialisation	Interpersonal relationships	23 (8, 11–47)	0:9	23 (7, 8–42)	0:9	0.449
	Play and leisure	12 (8, 2–32)	1:1	14 (6, 8–36)	1:3	0.172
	Coping skills	3.5 (8, 0–29)	0:7	10 (7, 6–17)	2:1	0.031
Motor skills	Gross	12 (7, 3–79)	0:7	47 (7, 4–67)	1:8	0.096
	Fine	16.5 (8, 1–54)	1:3	34 (7, 12–64)	3:0	0.093

ECBI behaviour *intensity* and *problem* scores were significantly lower for children with MPS III than ID (Table 5), with large effect sizes, (*U* = 9, *z* = -2.199, *r* = -0.568, *p =* 0.028) and (*U* = 6.5, *z* = -2.086, *r* = -0.578, *p =* 0.037), respectively*.* The behaviour *intensity* score for children with ID exceeded clinical threshold (131) for problem behaviour while the MPS III score does not. The MPS III group had significantly lower scores on the *irritability* domain (*U* = 12.5, *z* = -2.025, *r* = -0.506, *p* = 0.043) and on the *current understanding* (*U* = 11, *z* = -2.345, *r* = -0.569, *p* = 0.019) and *current expression* subdomains (*U* = 6, *z* = -2.848, *r* = -0.691, *p* = 0.004) of the SBRS. Of the MPS III group, 90% were reported to have shown better comprehension and expressive communication skills in the past, compared to 28.5% of the ID group. Of the children with MPS III, 60% had sleep problems (40% severely disrupted), 90% were no longer continent, 10% had behavioural problems or over-activity, 50% no longer walked and 60% were unresponsive most of the time.

**Table 5:** **Behaviour-related domain scores (10–15 year olds)**

	MPS III median ( ***N***, range)	ID median ( ***N***, range)	***p***value
ECBI			
Intensity score	76 (8, 36–174)	155 (7, 74–201)	0.028
Problem score	1 (7, 0–21)	14.5 (6, 1–29)	0.037
ABC			
Irritability	2 (9, 0–24)	23 (7, 1–40)	0.043
Lethargy	7 (9, 2–28)	7 (7, 4–27)	0.915
Stereotypy	2 (9, 0–14)	5 (7, 8)	0.183
Hyperactivity	7 (9, 2–28)	23 (7, 1–41)	0.152
Inappropriate speech	0 (9,0- 5)	4 (7, 0–12)	0.054
ABC total score	26 (9, 6–80)	54 (7, 11–106)	0.081
**SBRS**			
Current understanding	10 (10, 2–38)	33 (7, 14–41)	0.019
Past understanding	28 (9, 16–42)	42 (2, 4–20)	0.056
Current expression	2 (10, 0–12)	11 (7, 5–23)	0.004
Past expression	12 (9, 6–24)	12 (2, 4–20)	0.813
Orality	20 (10, 2–36)	15 (7, 0–26)	0.243
Body movements	10 (10, 4–30)	10 (7, 0–23)	0.590
Interactions with objects	11 (10, 3–19)	8 (7, 0–15)	0.240
Activity and routines	14 (10, 4–30)	20 (7, 6–26)	0.845
Emotional function	6 (10, 0–13)	5 (7, 2–14)	0.883
Safety consciousness	12 (10, 0–18)	8 (7, 3–14)	0.352
Fearfulness	28 (10, 12–35)	16 (7, 8–36)	0.405
Social interaction	13.5 (10, 2–22)	17 (7, 8–25)	0.282
Eye contact	3 (10, 0–8)	6 (7, 0–10)	0.258
Emotional engagement	7.5 (10, 4–14)	6 (7, 2–13)	0.257
Comfort seeking	10 (10, 8–14)	11 (7, 0–20)	0.428
Attention	12 (10, 6–18)	11 (7, 3–18)	0.883
Self-control/compliance	6 (10, 0–16)	10 (7, 4–18)	0.281
Mood, anger and aggression	5.5 (10, 0–42)	19 (7, 2–33)	0.117
Self-gratification	0 (10, 0–2)	2 (7, 0–5)	0.217

## Discussion

In the 2–9-year age range, gross motor skills were the only adaptive skills that differentiated between the MPS III and ID groups. In the 10–15-year age group, the ID group showed significantly more advanced adaptive skills than the MPS III group in all areas of daily living skills, written communication and coping skills and in current understanding and current expression. Thus, level of disability increased with age in the MPS III group, while the ID group acquired new skills with age, possibly accounting for the age-related decrease in challenging behaviour in MPS III as they lose physical and cognitive skills and are less able to actually perform such behaviour. Although such behavioural problems are a feature of the middle phase of MPS III, the high frequency is not in itself phenotypic and may be associated with ID level. Middle phase children with MPS III displayed significantly more behaviours relating to hyperactivity, orality, body movements, interactions with objects and inattention than the control group, but given the poor internal consistency of the *interactions with objects* domain on the SBRS, this finding should be viewed with caution. Such behaviours may be part of the behavioural phenotype of the middle phase of MPS III, but this requires further investigation [36, 37]. In the late phase MPS III group, few behaviours remained problematic; possibly, parents were used to managing higher levels of challenging behaviour in the middle phase and/or because the reduction in challenging behaviour corresponded to the inevitable physical and cognitive deterioration—one parent remarked that they wished their child was still able to display challenging behaviour.

The present findings confirm previous reports of behaviours relating to orality, unusual affect and hyperactivity in the middle phase of MPS III and add that they occur significantly more frequently compared to matched controls. A novel finding was of unusual body movements being phenotypic in the middle stage of MPS III. The previously reported high rates of challenging behaviour and physical aggression in MPS III were found to be no different from matched controls in the middle phase and are probably associated with the level of ID. Interestingly, although unusual/inappropriate affect were no more frequent compared to matched controls, they were displayed by children with MPS III throughout their lives and even after other behaviours had disappeared. Unlike previous research, this study did not examine ‘temper tantrums’ as these are poorly defined and subjective in report. This study found sleep disturbance to be a common problem in MPS III but with lower prevalence than previous studies, which with a parallel of sleep in MPS III that identified that the quantity of night-time sleep in children with MPS III was not significantly different from typically developing children [23].

This study was limited by the small sample size and grouping of MPS III subtypes. It is possible that the within-group variability found in this study could be accounted for by genetic subtype. As MPS III subtypes are genetically distinct, the findings of this study can only be described as preliminary and identify areas to focus future research. A larger sample size would also show fewer outliers, as was the case in the late phase MPS III sample where there was an outlier in terms of ability, although this did not substantially affect the findings, and it is likely that this was a case of the MPS III B mild phenotype and thus indicative of the heterogeneous presentation of MPS III [15, 14].

The SBRS is a relatively under-developed measure that requires further work to improve its psychometric properties, and therefore, the data derived from the SBRS should be treated with caution.

### Clinical implications

The present findings indicate that families with children with MPS III may benefit from a different type of support service, in addition to their medical treatment, in the middle phase compared to the late phase of the disorder. In the middle phase, needs associated with hyperactivity and behavioural concerns could be met by community learning disability services, while issues relating to deterioration and loss of skills and end-of-life care in the late phase may be best met by paediatric psychology services, although the heterogeneity in individual presentation means the age at which these needs change will vary. In the middle phase, the behavioural problems related to inattention and hyperactivity may benefit from the same type of behavioural interventions as children of a similar developmental level diagnosed with Attention Deficit Hyperactivity Disorder (ADHD). Additionally, a parallel study found that parents of children with MPS III experience similar levels of stress to those with a child with ID [23]. The National Institute for Clinical Excellence (NICE) guidelines recommend parenting groups as the primary intervention for children with ADHD and ID with subsequent individual parenting skills interventions if necessary [38]. As behavioural interventions are effective in MPS III [5], parenting interventions could be developed for parents of children with MPS III which could address both managing behavioural issues and coping with the progressive, terminal prognosis of MPS III.

## Conclusions

Although this study was predicated on a biological basis for the behaviour of children with MPS III, the complex relationship between environment, biology, learning and personal factors must be considered given that social context [39], physical environment and triggers [40] and effect of personal characteristics on phenotypic behaviour [41] are demonstrably important when examining behaviour in other genetic syndromes. Examination of differences in behavioural presentation between the genetic subtypes of MPS III would also inform the understanding of the genotype-phenotype relationship in MPS III, but this may be difficult within a UK sample and might require international recruitment, possibly utilising on-line data collection.

No single questionnaire in this study captured the behavioural phenotype or was completed by parents exactly according to guidelines, and the present findings should inform further development of existing and novel questionnaire-based measures for use with this small but important population [9, 10]. Moreover, given the progressive nature of MPS III coupled with the evident phenotypic heterogeneity, future research could use more naturalistic methodologies with an emphasis on describing the progressive nature of the disorder rather than on mapping evident differences.
